# Current Concepts - Congenital Scoliosis

**DOI:** 10.2174/1874325001711010337

**Published:** 2017-04-28

**Authors:** Agnivesh Tikoo, Manish K. Kothari, Kunal Shah, Abhay Nene

**Affiliations:** I (FNB Spine Surgery) Wockhardt Hospitals, 1877, Dr. Anand Rao Nair Road, Mumbai Central (E), Mumbai- 400 011, India; 2 (MS Ortho) Wockhardt Hospitals, 1877, Dr. Anand Rao Nair Road, Mumbai Central (E), Mumbai- 400 011, India

**Keywords:** Early onset scoliosis, Congenital scoliosis, Convex epiphysiodesis, Growth rods, Hemivertebrectomy, Scoliosis

## Abstract

**Background:**

Congenital scoliosis is one of the ‘difficult to treat’ scenarios which a spine surgeon has to face. Multiple factors including the age of child at presentation, no definite pattern of deformity and associated anomalies hinder the execution of the ideal treatment plan. All patients of congenital scoliosis need to be investigated in detail. X rays and MRI of spine is usually ordered first. Screening investigations to rule out VACTERL (Visceral, Anorectal, Cardiac, Tracheo-esophageal fistula, Renal and Lung) abnormalities are required. They are cardiac echocardiography and ultrasonography of abdomen and pelvis. CT scan is required to understand the complex deformity and is helpful in surgical planning.

**Methods:**

A comprehensive medical literature review was done to understand the current surgical and non surgical treatment options available. An attempt was made to specifically study limitations and advantages of each procedure.

**Results:**

The treatment of congenital scoliosis differs with respect to the age of presentation. In adults with curves more than 50 degrees or spinal imbalance the preferred treatment is osteotomy and correction. In children the goals are different and treatment strategy has to be varied according to the age of patient. A single or two level hemivertebra can easily be treated with hemivertebra excision and short segment fusion. However, more than 3 levels or multiple fused ribs and chest wall abnormalities require a guided growth procedure to prevent thoracic insufficiency syndrome. The goal of management in childhood is to allow guided spine growth till the child reaches 10 - 12 years of age, when a definitive fusion can be done. The current research needs to be directed more at the prevention and understanding the etiology of the disease. Till that time, diagnosing the disease early and treating it before the sequels set in, is of paramount importance.

**Conclusion:**

The primary aim of treatment of congenital scoliosis is to allow the expansion of chest and abdominal cavity, while keeping the deformity under control. Various methods can be categorized into definitive (hemivertebrectomy) or preventive (guided growth). Casting, Growth rods, Convex Epiphysiodesis are all guided growth measures. The guided growth procedure either ‘corrects the deformity’ or will have to be converted to a final fusion surgery once the child completes the spinal growth which is preferably done around 10 - 12 years of age. Future directions should aim at genetic counselling and early detection.

## INTRODUCTION

SRS has defined the Early Onset Scoliosis as a lateral curve of the spine that is diagnosed before age 10. Early onset scoliosis can be sub classified to include congenital scoliosis, idiopathic infantile and juvenile scoliosis. EOS (Early Onset Scoliosis) also included the neuromuscular and syndromic scoliosis presenting before age 10. Whatever is the etiology, treatment goals remain common. The alveoli grow in number till 8 years of age and after that there is increase in the size of alveoli till skeletal maturation. Any volume depleting deformity of chest wall in early age will lead to poor lung maturation and severely affect the respiratory system of an individual. This is called *Thoracic Insufficiency Syndrome.*

## CONGENITAL SCOLIOSIS HOW IS IT DIFFERENT?

The term “congenital scoliosis” refers to a spinal deformity caused by vertebrae that have not formed properly. The defect occurs early in life and no cause has been definitely associated with the condition. However, it has serious consequences on spinal growth. A detailed perinatal history is required to differentiate it from other causes of early onset scoliosis. More often than not, the vertebral defect/ scoliosis gets detected on a chest X-ray ordered for chest evaluation. Whether the patient presents with a hump or the defect is visualized on a chest x ray done, unlike idiopathic scoliosis which can be observed, a complete investigation in warranted. MRI of the spine must be ordered to look for associated cord abnormalities like tethering, Split Cord Malformations, Closed dysraphisim.

Screening of other congenital anomalies especially of renal, cardiac and lung should be done. Tracheo-esophageal fistula can present as failure to thrive. Anorectal anomalies are detected in neonatal period only. CT scan is helpful for the surgical planning of patient and also helps in knowing whether the split cord malformation septum is bony or fibrous. Congenital Vertebral defects may present as scoliosis, kyphoscoliosis or pure kyphosis

The etiology of congenital scoliosis is not properly understood. Failure of formation/ segmentation during somitogenesis has been proposed. Notch, FGF and Wnt signaling pathways have been associated with the process of somitogenesis [1]. Associations with Carbon Monoxide exposure, maternal diabetes and anti-epileptic drugs has also been proposed [2]. The presence of congenital scoliosis in twins and siblings is not well understood. Cases of congenital scoliosis in non-identical [3] and identical twins [4] have been reported. It is more common in girls than boys. Familial incidence is about 1 - 5% [5, 6].

The progression of a congenital deformity would depend on (a) the type of anomaly, (b) the site, and (c) the spinal growth potential of the individual. Left untreated, 85% of the patients would have final curves greater than 41 degrees [7]. Winter studied 234 patients for the natural history and progression of congenital scoliosis. Winter’s classification is widely accepted and followed classification [8, 9]. This was further modified and enlarged by McMaster classifying these defects into 4 types [10]:

Type I - failure of formation

Type II - failure of segmentation

Type III - combination of an anterolateral unsegmented bar and one or more contralateral posterolateral quadrant vertebrae.

Type IV - unclassified.

Type III-kyphosis/ kyphoscoliosis has the highest rate of progression followed by type I. The lumbar or lumbosacral hemivertebrae result in more severe deformity because they are difficult to compensate by the rest of the spine to maintain truncal balance. The type III vertebral defects have higher rates of progression than other subtypes.

## RADIOGRAPHIC ASSESSMENT EVERY PATIENT OF ONGENITAL SCOLIOSIS SHOULD HAVE AN MRI

**Plain X-Rays** are helpful for a gross overview of the deformity. Sometimes it is difficult to understand the exact nature of the malformation on standard AP and Lateral spine x rays. A CT helps in delineating the anomaly better. The x rays are used to assess the flexibility of the compensatory curves and measurement of Cobb angle for comparisons on follow up. A push prone view (by pressing on the kyphosis while x-ray is being taken) especially done in adult cases is useful to assess the rigidity of a kyphotic deformity is, which helps in surgical planning.

**CT Scan** with reconstruction images help to understand the complex vertebral deformities. Any bony septum within the canal can also be identified. 3D reconstruction can mark out posterolateral hemivertebrae very well. The use of 3D CT data to define lung volumes in patients who were too young for pulmonary function tests has been described. Improvement in lung function following expansion thoracoplasty has also been documented using 3D CT [11].

**MRI** is the basic part of assessment of congenital scoliosis to look for spinal cord anomalies, tethering and spinal dysraphism. Craniovertebral screeening should also be done to look for any syrinx or a Chairi malformation.

## TREATMENT

The aim of treatment in congenital scoliosis is having a stable balanced spine and arrest the progression of deformity while allowing spinal growth. Non-surgical treatment is seldom effective but nevertheless has some role to play in selected cases

Block vertebra (type II anomaly) and Nonsegmented vertebra (Type I anomaly) especially if they are at one level only, remain static and just need observation. Balanced hemivertebrae and thoracolumbar hemivertebra (<50 degrees) can be observed over a period of time to document progression before treatment is planned. Primary objective is to avoid a thoracic insufficiency syndrome and maintain spinal balance.

## Non Surgical Treatment - A Time Buying Strategy

It is very difficult to control the progression of deformity in a growing spine. Casting and bracing are two possible options but their use is as a time buying tool to delay the primary surgery especially in younger children.

Bracing is seldom effective for the scoliosis. Compliance of bracing is difficult to achieve and deterioration in an already decompensated pulmonary function can be catastrophic.

At the time of presentation, few cases of congenital scoliosis are too small to allow pedicle screw fixation. On the other hand, they have significantly larger curves and can’t be neglected. Serial casting in small children is again coming up as a time buying strategy to delay the definitive surgery by upto 2 years [12]. Fletcher [13] retrospectively studied 29 patients with syndromic, neuromuscular, or congenital scoliosis or were older than 2.5 years with an idiopathic scoliosis measuring >50 degrees. From the time first cast was applied, surgery was postponed by 39 ± 25 months. The authors concluded that serial casting is a viable alternative to surgical growth sparing techniques in moderate-to-severe early-onset scoliosis and may help delay eventual surgical intervention.

## Surgical Treatment Options

All the studies focusing on the congenital scoliosis have clearly pointed out that without treatment the outcome usually is unacceptable. Of the untreated cases, only 10% have a curve of 20 degrees or less, and 64% have curves that are greater than 40 degrees [14]. As the age progresses, the compensatory deformities become more structural. Children who were operated before 5 years attained better correction [15].

Various surgical treatment modalities that have been described are *in-situ* posterior spinal fusion, combined anterior and posterior *in-situ* spinal fusion, convex hemiepiphysiodesis, hemivertebra excision, guided growth procedures like growing rods (Fig. **2**) and VEPTR. Hemivertebra excision is safe and a effective procedure for a single level congenital vertebral defect. The results are better when surgery is done early [16, 17].

**Fig. (1):**
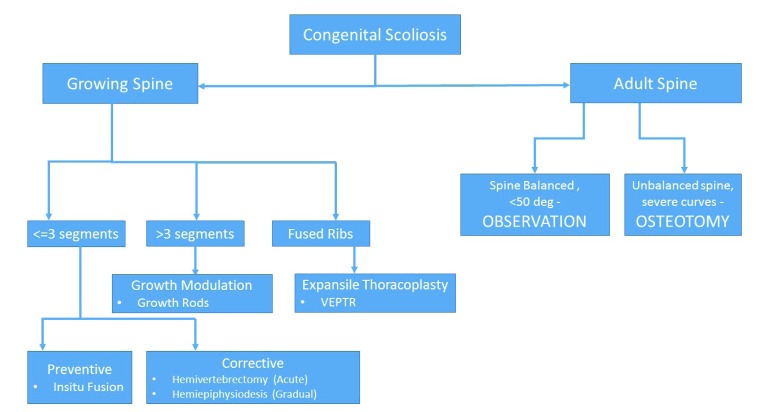
A guide to surgical decision making in Congenital Scoliosis.

**Fig. (2):**
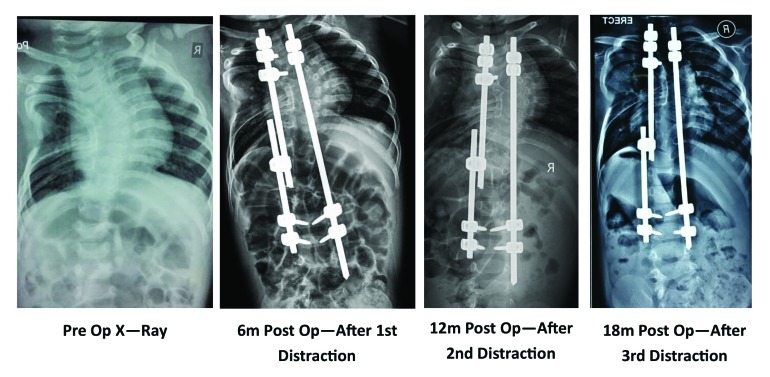
18 month old male child with congential scoliosis with multiple fused ribs and concave bar on left dorsal spine. Note the opening up of chest cavity on left side in serial post op distractions. Rod was not fully tightened to right lower screws to allow it to slide freely.

The treatment strategy for a congenital scoliosis may change with the age of patient and the type of deformity. As a general rule, for a single hemivertebra, excision and short segment fusion done at an early age (3-5 years) gives the best results. However, for long (>4) unfused segments the strategy changes to guided growth procedure allowing chest expansion and spinal growth, and definitive fusion at a later date preferably after 10 - 12 years of age. A guide to the surgical decision making is given in (Fig. **1**).

## Posterior *In-situ* Fusion

In this method, posterior fusion of spine was performed from end vertebra to end vertebra of the curve. The most ideal indication of posterior in situ fusion is a small curve (30 - 40 degrees) in a small child (2-3) years with a type 2 segmentation defect or a fused concave bar extending less than 3 segments. This prevents any further deformity, allowing the remaining spine to grow normally. Fusion of 4 or more segments caused a severe decrease in lung function as compared to healthy individuals [18]. Winter reported that even after fusion, there is ‘bending of fusion mass’ posteriorly, which causes late deterioration of scoliosis after posterior fusion in about 14% of the cases [19]. Kesling reported this incidence to be 15% [20]. To prevent this late deterioration of the curve, hemivertebra excision is considered a more definitive and better procedure.

## Anterior Hemivertebra Excision

The earliest anterior excision of hemivertebra was reported by Royle in 1928 [21]. This was followed later by Compere [22] and, Von Lackum and Smith in lumbar spine [23]. Wiles performed the operation in the thoracic spine using a posterolateral approach by resecting ribs in two children [24]. However, the children developed kyphosis because of unrestricted posterior growth. In addition the procedure was difficult in kyphoitic cases. Posterior fusion was added after the anterior hemivertebrectomy to avoid kyphosis and allow instrumentation for better correction.

## Two Stage Excision

Leatherman and Dickson described excision of the hemivertebrae (anterior and posterior closing wedge osteotomy) [25, 26]. The two stage procedure was performed 5-7 days apart for risk of spinal cord ischemia. However, later the procedure was safely performed in a single stage.

## Single Stage Anterior-posterior Excision

In single stage excision, the anterior vertebral body, pedicle and discs were removed. In the second stage the posterior arch was removed and the convexity was closed and reduction achieved. This was initially supplemented by casting. Later with the introduction of instrumentation, the casting was not needed. Bradford and Boachie-Adje suggested that the procedure can be done in a single stage [27]. They described the procedure in seven patients, who underwent a single stage anterior and posterior arthrodesis achieving approximately 70% correction after an average follow-up of 45.6 months. Kokubun [28] and Leatherman [29] also showed single stage anterior-posterior hemivertebrectomy as a safe and efficacious procedure. Even though being single stage, there was a significant morbidity of anterior surgery involved and there were two separate incisions and surgeries.

## Posterior Only Hemivertebrectomy and Fusion (Figs. **3** and **4**)

Shono [30] in 2001 retrospectively studied 12 patients in the age group of 8 - 24 years with kyphoscoliosis caused by a single hemivertebra. Preoperative scoliosis averaging 49° was corrected to 18^0^ and kyphosis averaging 40° was corrected to 17°. They concluded that single stage posterior hemivertebrectomy was safe and effective in structural kyphoscoliotic deformity caused by a thoracic or thoracolumbar single hemivertebra. Late deterioration by crankshaft mechanism usually does not happen because the anterior growth plates do not have normal growth potential. Ruf and Harms [31] (2002) described the procedure in 21 consecutive cases and achieved significant correction in both the coronal and sagittal planes. Because of short segmental fusion, high stability, no need for an anterior approach, and low neurologic risk, they concluded the procedure to be safe and efficacious. They also recommended that surgery should be performed as early as possible to avert severe local deformities, to prevent secondary structural changes, and to avert extensive fusions.

Wang [32] retrospectively analyzed 60 patients divided equally in single stage anterior-posterior and posterior only group. The analysis was done to evaluate operation time, blood loss, degree of correction of the main curve/segmental curve/ kyphosis, the average hospital stay, and complications. They found that mean operation time, blood loss, and hospital stay were significantly less in the posterior only group. The average correction rate of the main curve/segmental curve/ kyphosis of the anterior-posterior group was marginally better (*p*>.05). However, they reported a complication rate was 6.7% in the former group vs. 10% in the posterior group (*p*>.05) 2/3^rd^ of which were radiculopathies.

Yaszay [33] in a multicenteric trial compared three modalities *i.e*. hemi-epiphysiodesis or in situ fusion, instrumented fusion without hemivertebra excision, and instrumented hemivertebra excision. The author concluded that posterior hemivertebra resection in younger patients resulted in a better correction (percentage) than the other two techniques, although it also was associated with higher complication rate. However, despite some of its limitations posterior resection of hemivertebrae with transpedicular instrumentation is a safe and promising procedure [34].

## Convex Epiphysiodesis

Convex epiphysiodesis is a slow correction procedure. The procedure is done in two stages. First the anterior vertebral convex side of discs are removed which are packed with bone chips taken from the rib excised during approach. It is important to remove two discs above and below the hemivertebra otherwise the correction will not be possible. Now, the anterior part of surgery can also be done thoracoscopically. In second step, surgery the convex side facet joints and posterior elements are fused. However, the procedure needs careful follow up and patient may have to undergo additional surgeries if the epiphysiodesis is not able to control the progression. Very few reports and personal experiencies of different authors have been published on the subject. Using convex epiphysiodesis with concave distraction has also been suggested [35].

## Growth Rods

 The concept of growth rods has developed slowly over a period of time. Harrington [36], Luque [37], and Moe [38] all used the ‘growing’ concept in different cases. The growth rods can be single or dual. A single growth rod was put on the concavity of the curve and distracted at regular intervals. This may be supplemented with a convex epiphysiodesis. Akbarnia [39] published their results of submuscular dual-growing rods in the management of progressive scoliosis in 2000. The short term results in congenital scoliosis are promising however, long term results are still awaited. MAGEC is a magnetically controlled growth rod which is currently being used for EOS. However, its usefulness in congenital scoliosis is not yet proved. However, theoretically it looks promising because it avoids the multiple invasive procedures for distraction.

## Thoracic Insufficiency Syndrome, Thoracoplasty and VEPTR

Thoracic Insufficiency syndrome is defined as the inability of the thorax to support normal respiration or lung growth. The concept was highlighted by Campbell who has pioneered the art of expansile thoracoplasty [40]. The procedure involves osteotomizing the concave fused ribs and distracting them progressively over a period of time to simulate chest cavity growth. Size of implants and small ribs may make it is difficult to be used in children less than 1 year old. The implant can be used as rib to rib, spine to rib or ilium to rib construct. The serial distractions are done by a minimally invasive technique. In 2004, Campbell reviewed the 27 cases of opening wedge thoracotomy and VEPTR. Serial CT scans showed the improvement in space available for lungs. Only three patients were old enough to cooperate for the baseline PFT. However serial PFTs showed mean 47% of predicted vital capacity at last follow up. The maximum improvement was seen in patients treated before age of 2 years.

## Osteotomies in Adult

Congenital scoliosis presenting in adulthood (after completion of spinal growth) requires osteotomy due to the rigid deformity. The deformity is managed on the principles of adult deformity. Pedicle subtraction osteotomy and Vertebral Column Resection being the procedures of choice based on each case.

## CONCLUSION

Congenital Scoliosis is one of the most difficult type of Early Onset Scoliosis to manage. The vertebral defect can lead to a significant deformity and restriction of lung growth resulting in Thoracic Insufficiency Syndrome. The foremost aim of treatment is to allow the expansion of chest and abdominal cavity, and keeping the deformity under control. The guided growth can be converted to a final fusion surgery once the child completes the spinal growth. This is preferably done around 10 - 12 years of age.

### Future Directions

The future of congenital scoliosis lies in the early diagnosis of the disease and preventing its consequences especially thoracic insufficiency syndrome. Three-dimensional understanding of deformity and chest cavity will help better in achieving the goals.

We also need to look at the genetic aspect of the Congenital Scoliosis to know how to prevent it. May be in future we may be able to treat the vertebral anomaly during fetal life and avoid the untoward toll and consequences that the disease has on its patients despite best of treatment efforts.

## Figures and Tables

**Fig. (3):**
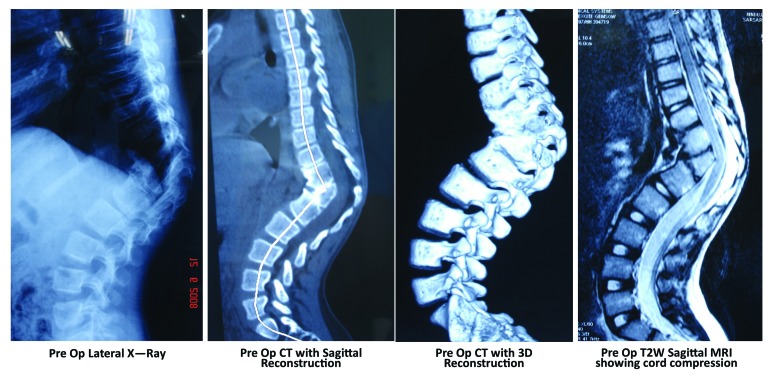
13 year old female patient with congential kyphosis with progressive paraparesis. Note the presence of extra pedicle with anterior aplasia and toppling in CT Scan. MRI shows cord compression.

**Fig. (4):**
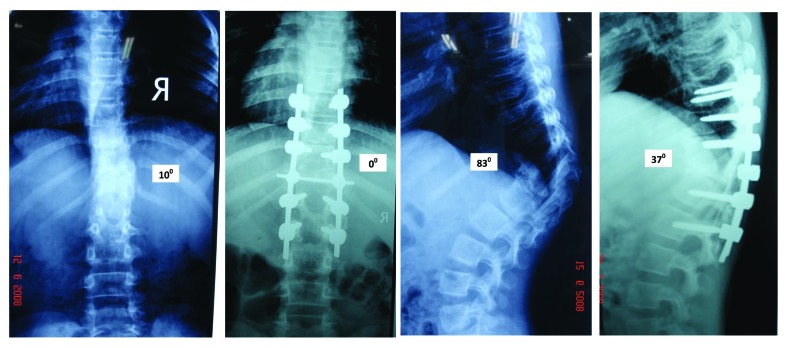
Pre Op and post Op X rays of the same patient (Fig. **3**).

## References

[1] Giampietro P.F., Dunwoodie S.L., Kusumi K., Pourquié O., Tassy O., Offiah A.C., Cornier A.S., Alman B.A., Blank R.D., Raggio C.L., Glurich I., Turnpenny P.D. (2009). Progress in the understanding of the genetic etiology of vertebral segmentation disorders in humans.. Ann. N. Y. Acad. Sci..

[2] Farley F.A., Hall J., Goldstein S.A. (2006). Characteristics of congenital scoliosis in a mouse model.. J. Pediatr. Orthop..

[3] Greenwood D., Bogar W. (2014). Congenital scoliosis in non-identical twins: case reports and literature review.. J. Can Chiropr Assoc..

[4] Winter R.B., Lonstein J.E. (2010). Scoliosis secondary to a hemivertebra: seven patients with gradual improvement without treatment.. Spine.

[5] Shahcheraghi G.H., Hobbi M.H. (1999). Patterns and progression in congenital scoliosis.. J. Pediatr. Orthop..

[6] Purkiss S.B., Driscoll B., Cole W.G., Alman B. (2002). Idiopathic scoliosis in families of children with congenital scoliosis.. Clin. Orthop. Relat. Res..

[7] McMaster M., Ohtsuka K. (1982). The natural history of congenital scoliosis: a study of 251 patients.. J. Bone Joint Surg..

[8] Winter R.B., Moe J.H., Eilers V.E. (1968). Congenital scoliosis: A study of 234 patients treated and untreated: Part I. Natural history.. J. Bone Joint Surg. Am..

[9] Winter R.B., Moe J.H., Wang J.F. (1973). Congenital kyphosis. Its natural history and treatment as observed in a study of one hundred and thirty patients.. J. Bone Joint Surg. Am..

[10] McMaster M.J., Singh H. (1999). Natural history of congenital kyphosis and kyphoscoliosis. A study of one hundred and twelve patients.. J. Bone Joint Surg. Am..

[11] Batra S., Ahuja S. (2008). Congenital scoliosis: management and future directions.. Acta Orthop. Belg..

[12] Demirkiran H.G., Bekmez S., Celilov R., Ayvaz M., Dede O., Yazici M. (2015). Serial derotational casting in congenital scoliosis as a time-buying strategy.. J. Pediatr. Orthop..

[13] Fletcher N.D., McClung A., Rathjen K.E., Denning J.R., Browne R., Johnston C.E. (2012). Serial casting as a delay tactic in the treatment of moderate-to-severe early-onset scoliosis.. J. Pediatr. Orthop..

[14] Nasca R.J., Stilling F.H., Stell H.H. (1975). Progression of congenital scoliosis due to hemivertebrae and hemivertebrae with bars.. J. Bone Joint Surg. Am..

[15] Marks D.S., Sayampanathan S.R., Thompson A.G., Piggott H. (1995). Long-term results of convex epiphysiodesis for congenital scoliosis.. Eur. Spine J..

[16] Bergoin M., Bollini G., Taibi L., Cohen G. (1986). Excision of hemivertebrae in children with congenital scoliosis.. Ital. J. Orthop. Traumatol..

[17] Bradford D.S., Boachie-Adjei O. (1990). One-stage anterior and posterior hemivertebral resection and arthrodesis for congenital scoliosis.. J. Bone Joint Surg. Am..

[18] Vitale M.G., Matsumoto H., Bye M.R., Gomez J.A., Booker W.A., Hyman J.E., Roye D.P. (2008). A retrospective cohort study of pulmonary function, radiographic measures, and quality of life in children with congenital scoliosis: an evaluation of patient outcomes after early spinal fusion.. Spine.

[19] Winter R.B., Moe J., Lonstein J.E. (1984). Posterior spinal arthrodesis for congenital scoliosis: an analysis of 290 patients 5 to 19 years old.. J. Bone Joint Surg. Am..

[20] Kesling K.L., Lonstein J.E., Denis F., Perra J.H., Schwender J.D., Transfeldt E.E., Winter R.B. (2003). The crankshaft phenomenon after posterior spinal arthrodesis for congenital scoliosis: a review of 54 patients.. Spine.

[21] Royle N.D. (1928). The operative removal of an accessory vertebra.. Med. J. Aust..

[22] Compere E.L. (1932). Excision of hemivertebrae for correction of congenital scoliosis: Report of two cases.. J. Bone Joint Surg..

[23] Von Lackum W.H., Smith A.D. (1933). Removal of vertebral bodies in the treatment of scoliosis.. Surg. Gynecol. Obstet..

[24] Wiles P. (1951). Resection of dorsal vertebrae in congenital scoliosis.. J. Bone Joint Surg. Am..

[25] Holte D.C., Winter R.B., Lonstein J.E., Denis F. (1995). Excision of hemivertebrae and wedge resection in the treatment of congenital scoliosis.. J. Bone Joint Surg. Am..

[26] Leatherman K.D., Dickson R.A. (1979). Two-stage corrective surgery for congenital deformities of the spine.. J. Bone Joint Surg. Br..

[27] Bradford D.S., Boachie-Adjei O. (1990). One-stage anterior and posterior hemivertebral resection and arthrodesis for congenital scoliosis.. J. Bone Joint Surg. Am..

[28] Kokubun S., Sakurai M., Rijial K.P. (1991). Operative technique of one-stage anterior and posterior excision of hemivertebra.. J. Jpn. Scoliosis Soc..

[29] Leatherman K.D. (1973). The management of rigid spinal curves.. Clin. Orthop. Relat. Res..

[30] Shono Y., Abumi K., Kaneda K. (2001). One-stage posterior hemivertebra resection and correction using segmental posterior instrumentation.. Spine.

[31] Ruf M., Harms J. (2002). Hemivertebra resection by a posterior approach: innovative operative technique and first results.. Spine.

[32] Wang L., Song Y., Pei F., Liu L., Liu H., Kong Q., Li T., Zeng J. (2011). Comparison of one-stage anteroposterior and posterior-alone hemivertebrae resection combined with posterior correction for hemivertebrae deformity.. Indian J. Orthop..

[33] Yaszay B., OBrien M., Shufflebarger H.L., Betz R.R., Lonner B., Shah S.A., Boachie-Adjei O., Crawford A., Letko L., Harms J., Gupta M.C., Sponseller P.D., Abel M.F., Flynn J., Macagno A., Newton P.O. (2011). Efficacy of hemivertebra resection for congenital scoliosis: a multicenter retrospective comparison of three surgical techniques.. Spine.

[34] Basu S., Tikoo A., Malik F.H., Ghosh J.D., Jain M., Sarangi T. (2016). Single and multiple level one stage posterior hemivertebrectomy and short segment fixation: experience with 22 cases and comparison of single *vs*. multilevel procedures with minimum 2-year follow-up.. Asian Spine J..

[35] Alanay A., Dede O., Yazici M. (2012). Convex instrumented hemiepiphysiodesis with concave distraction: a preliminary report.. Clin. Orthop. Relat. Res..

[36] Harrington P.R. (1962). Treatment of scoliosis. Correction and internal fixation by spine instrumentation.. J. Bone Joint Surg. Am..

[37] Luque E.R. (1982). Paralytic scoliosis in growing children.. Clin. Orthop. Relat. Res..

[38] Moe J.H., Kharrat K., Winter R.B., Cummine J.L. (1984). Harrington instrumentation without fusion plus external orthotic support for the treatment of difficult curvature problems in young children.. Clin. Orthop. Relat. Res..

[39] Akbarnia B.A. (2000). Instrumentation with limited arthrodesis for the treatment of progressive early-onset scoliosis.. Spine: State Art Rev..

[40] Campbell R.M., Smith M.D., Mayes T.C., Mangos J.A., Willey-Courand D.B., Kose N., Pinero R.F., Alder M.E., Duong H.L., Surber J.L. (2003). The characteristics of thoracic insufficiency syndrome associated with fused ribs and congenital scoliosis.. J. Bone Joint Surg. Am..

